# Supplementary material for: The Vaccination Concerns in COVID-19 Scale (VaCCS): Development and validation
Source: PLoS One. 2022 Mar 14;17(3):e0264784. doi: 10.1371/journal.pone.0264784 (PMC8920277; doi:10.1371/journal.pone.0264784)
Supplement: S1 File — (DOCX) [file pone.0264784.s001.docx]

**S1 File. Coding of participant responses and theme extraction in Phase 1 of the study.**

| **Theme** | **Questions and Codes** | **N** |
| --- | --- | --- |
|  | **“When you hear the word ‘COVID-19 vaccination, what comes to mind?”** |  |
| Behavior | I'll get the vaccine | 7 |
|  | I'm not getting it | 6 |
|  | Uncertain about getting it | 9 |
| Protection | Protection against COVID | 21 |
| Barriers | Difficult to get the vaccine | 7 |
| Emotions | Fear of side effects | 11 |
|  | Relief | 5 |
| Normality | Getting back to normal | 7 |
| Safety | Provides safety | 7 |
| Trust | It’s a scam | 5 |
| Development | Developed too quickly | 7 |
|  |  |  |
|  | **“What specific benefits does the COVID-19 vaccinations have, in your view?”** |  |
| None | None | 10 |
| Normality | Can go back to normal | 9 |
|  | Can freely go out and travel again | 8 |
| Protection | Protection against COVID | 27 |
|  | Protection for others | 9 |
| Efficacy | Provides immunity to virus | 8 |
|  | Reduces number of deaths | 12 |
|  | Reduces COVID symptoms if contracted | 8 |
|  | Stops the spread of COVID | 13 |
|  | Decrease chance of contracting COVID | 9 |
| Safety | Will keep me and others safe | 10 |
| Trust | Unsure of benefits | 6 |
| Emotions | Peace of mind | 8 |
|  |  |  |
|  | **“What specific concerns and/or worries do you have about the COVID-19 vaccinations?”** |  |
| None | None | 17 |
| Development | Developed too quickly | 8 |
|  | Not tested on enough people | 7 |
| Efficacy | Questions over effectiveness | 15 |
| Trust | Lack of trust in the vaccine | 6 |
| Safety | You will get the virus from the vaccination | 6 |
|  | Unsure of its safety | 7 |
|  | Side effects | 50 |
|  |  |  |
|  | **“What do you think about the scientific evidence for the safety and effectiveness of the COVID-19 vaccinations?”** |  |
| Trust | Believable and trustworthy | 19 |
|  | Sufficient / good enough | 27 |
|  | Trust in the authorities | 5 |
|  | Uncertain to trust the evidence | 27 |
| Efficacy | It is proven to be safe and effective | 9 |
| Development | Hasn’t been enough studies / not enough evidence | 30 |
|  |  |  |
|  | **“How do the important people in your life feel about the COVID-19 vaccinations?”** |  |
| Social influences | All for it | 25 |
|  | They agree with me | 8 |
| Social influences | Think it’s a good thing to do | 12 |
|  | Think it is necessary and important | 13 |
| Social influences | They will get it | 20 |
|  | They won’t get it | 13 |
|  | They are reluctant | 9 |
|  | Mixed thoughts on getting it | 9 |
| Emotions | Scared of side-effects | 13 |
| Trust | Don't trust it yet | 8 |
