# Supplementary material for: The Vaccination Concerns in COVID-19 Scale (VaCCS): Development and validation
Source: PLoS One. 2022 Mar 14;17(3):e0264784. doi: 10.1371/journal.pone.0264784 (PMC8920277; doi:10.1371/journal.pone.0264784)
Supplement: S2 File — (DOCX) [file pone.0264784.s002.docx]

**S2 File. Search syntax.**

OVID - 34 records retrieved

vaccin*.ti and (review.ti or meta-analysis.ti) AND (scale.ab or scale.ti or measur*.ti or measur*.ab)

Embase - 400 records retrieved

(measur*:ti,ab,kw OR scale:ti,ab,kw) AND vaccin*:ti AND (review:ti OR 'meta analysis':ti)

Medline - 346 records retrieved

(AB scale OR AB measur*) AND TI vaccin* AND (TI review OR TI meta-analysis)

Web of Science - 358 records retrieved

(TI=(vaccin*) AND TI=(review OR meta-analysis) AND TS= (scale OR measur*)) AND LANGUAGE: (English) AND DOCUMENT TYPES: (Article OR Review)
