# Supplementary material for: The Vaccination Concerns in COVID-19 Scale (VaCCS): Development and validation
Source: PLoS One. 2022 Mar 14;17(3):e0264784. doi: 10.1371/journal.pone.0264784 (PMC8920277; doi:10.1371/journal.pone.0264784)
Supplement: S3 File — (DOCX) [file pone.0264784.s003.docx]

**S3 File. PRISMA flow chart.**

Abstracts excluded: 425

Reviews excluded: 13

Did not meet criteria: 11

No unique scales: 2

Vaccine belief measurement reviews: 3

Other reviews: 7

Manuscripts from review of reviews: 63

Additional identified manuscripts: 1

Records identified through database search:

448

Records identified through database search:

1138

Manuscripts included:

64

Review manuscripts included:

10

Full-text articles assessed for eligibility:

23

Items extracted:

561
