# Supplementary material for: The Vaccination Concerns in COVID-19 Scale (VaCCS): Development and validation
Source: PLoS One. 2022 Mar 14;17(3):e0264784. doi: 10.1371/journal.pone.0264784 (PMC8920277; doi:10.1371/journal.pone.0264784)
Supplement: S4 File — (DOCX) [file pone.0264784.s004.docx]

**S4 File. Extracted scale items and their sources.**

| Authors | Year | Extracted Items Sources |  |
| --- | --- | --- | --- |
| Nefta et al. | 2020 | Baldwin et al,. 2013 | Bodson et al., 2016 (No relevant items); Glen et al., 2015 (Items not reported); Kepka et al., 2015 (Items not reported); Colon-Lopez et al., (Items not reported); Marlow et al., 2009 (Items not reported) |
| Newman & Logie | 2010 | Crosby et al., 2004; Gagnon, 2000; Ritvo, 2003 | Newman et al., 2010 (Qualitative); Kakinami et al., 2008 (No relevant items); Liau et al., 1998 (Items not reported); Liau et al., 2000 (Items not reported); Newman et al., 2006 (No relevant items); Zimet et al., 1996 (Items not reported); Zimet et al., 1997 (Items not reported); Zimet et al., 1999 (Items not reported); Zimet et al., 2000 (No relevant items) |
| Allen et al. | 2010 | Barbin et al., 2006; Davis et al., 2004; Marlow et al., 2007a; Gerend et al,. 2007; Marlow et al., 2007b; Ogilvie et al., 2007; Gerend et al., 2008 | Ferris et al., 2007 (No relevant items); Boeher et al., 2003 (Extracted from another review); Kahn et al., 2008 (Items not reported); Donders et al., 2007 (No relevant items); Chan et al., 2007 (Items not reported); Kahn et al., 2003 (Items not reported); Dempsey, 2007 (Items not reported); Fazekas et al., 2008 (Items not reported); Lazcano-Ponce, 2001 (No relevant items); Giles et al., 2006 (No relevant items); Sauvageau et al. 2007 (Items not reported); Hausdorf et al., 2007 (Items not reported); Marshall et al., 2007- interview; Hoover et al., 2000 (Items not reported); Dinh et al., 2007 (Items not reported) |
| Larson et al. | 2015 | Gellin et al., 2000 | Opel et al., 2011 (Extracted from another review) |
| Larson et al. | 2018 | Kolar et al., 2015; Ronnerstrand, 2013; Lee et al., 2016; van der Weerd et al., 2011; Freimuth et al., 2017; Casiday et al., 2006; Quinn et al., 2017; Won et al., 2015; Sherer et al., 2016; Wu et al., 2008; Cheng et al., 2010; Freed et al., 2011; Fu et al., 2017; Moran et al., 2016; Raude et al., 2016; Wada et al., 2015 | Edmonds et al., 2011 (Extracted from another review); Chuang et al., 2015 (Items not reported); Taylor-Clarke et al., 2005 (Items not reported); Cooper et al., 2017 (No relevant items); Grabenstein et al., 2002 (Items not reported) |
| Herzog et al. | 2013 | Milliedge et at., 2003; Russell et al., 200; Gust et al., 2008; Salmon et al., 2008 | Clarke et al., 2006 (No relevant items); Wilson et al,. 2004 (Items not reported); Jungbauer-Gans et al., 2003 (Incorrect Language); Zimmerman et al., 2002 (No relevant items); Davis et al., 2003 (No relevant items); Davis et al,. 2007 (Items not reported); Taylor et al., 2002 (Items not reported); Gonik et al., 2000 (Items not reported); Schupfner et al., 2002 (Incorrect Language); Goodyear-Smith et al., 2009 (No relevant items) |
| Dyda et al. | 2020 | Obel et al., 2011; LaVail et al., 2013; Stefanoff et al., 2010 | Cassidey et al., 2006 (Extracted from another review); Umeh et al., 2018 (Extracted from another review). |
| Schellenberg & Crizzle | 2020 | Perinet et al., 2016; Carpiano et al., 2019 | Dube et al., 2016 (Extracted from another review); Gilbert et al., 2017 (Items not reported); MacDonald et al., 2014 (Items not reported); Greenberg et al., 2017 (No relevant items) |
| Schmid et al. | 2017 | Seale et al., 2010; Gargano et al., 2011; Kravos et al., 2014; Redelings et al., 2012; Liao et al, 2011; Galarce et al., 2011; Newcombe et al., 2014; Wu et al., 2014; de Perio et al., 2014; Porter et al., 2013 | Li et al., 2012 (Items not reported); Borjesson et al., 2014; Shahrabani et al., 2010 (Items not reported); Liao et al., 2014 (No relevant items); Kumar et al., 2012 (Items not reported); Ibuka et al., 2014 (Items not reported); Lau et al., 2012 (Items not reported); Offutt-Powell et al., 2014 (Items not reported); Shono et al., 2014 (Items not reported); Podlesek et al., 2011 (Items not reported); Steelfisher et al., 2015 (Items not reported); Renner et al., 2012 (Items not reported); Wiese-Posselt et al., 2006 (Items not reported); Suryadevara et al., 2014 (Items not reported); Villacorta et al., 2015 (Items not reported); Anne-Laure et al., 2014 (Items not reported); Chen et al., 2011 (Items not reported); Rudisill, 2013 (No relevant items); Naing et al., 2012 (Items not reported); Kiviniemi et al., 2011 (Items not reported); Huang et al., 2012 (Items not reported); Keller et al., 2014 (Items not reported); Prati et al., 2011 (No relevant items); Cole et al., 2015 (Items not reported); Han et al., 2016 (Items not reported); Leder et al., 2015 (No relevant items); Strelitz et al., 2015 (Extracted from another review) |
| Newman et al. | 2013 | Young et al., 2011; Crosby et al., 2011; Ferris et al., 2009 | Daley et al., 2010 (Items not reported); Daley et al., 2011 (Items not reported); Petrovic et al., 2011 (No relevant items); Blodt et al., 2012 (Items not reported); Newman et al., 2008 (Items not reported); Reiter et al., 2010 (Items not reported); Rieter et al., 2010 (Items not reported); Sauvageau et al. 2007 (Items not reported) |
| Chan et al. | 2012 | *No Unique Scales* | Dinh et al., 2007 (Items not reported); Dursun et al., 2009 (No relevant items); Gerend et al., 2007 (Extracted from another review); Madhivanan et al., 2009 (Qualitative); Marlow et al., 2007 (Extracted from another review); Marshall et al., 2007 (Qualitative); Ogilvie et al., 2007 (Extracted from another review); Sauvageau et al. 2007 (Items not reported); Tozzi et al., 2009 (No relevant items); Jaspan et al., 2008 (Items not reported) |
| Fajar & Harapan | 2017 | *No Usable Scales* | Hadisoemarto & Castro, 2013 (Items not reported); Harapan et al., 2016 (Items not reported) |
